# The lymphocyte-to-monocyte ratio is a superior predictor of overall survival compared to established biomarkers in HCC patients undergoing liver resection

**DOI:** 10.1038/s41598-018-20199-2

**Published:** 2018-02-07

**Authors:** Yu-Ting Yang, Jing-Hang Jiang, Hao-Jie Yang, Zhi-jun Wu, Ze-Min Xiao, Bang-De Xiang

**Affiliations:** 1 Department of Oncology, The First People’s Hospital of Changde City, Changde, 415000 China; 2 Department of Hepatobiliary Surgery, Affiliated Tumor Hospital of Guangxi Medical University, Nanning, 530021 China; 3 Department of General Surgery, Second People’s Hospital of Jing Men, Jingmen, 448000 China; 4 Department of General Surgery, The First People’s Hospital of Changde City, Changde, 415000 China

## Abstract

The aim of this study was to investigate the prognostic value of the lymphocyte-to-monocyte ratio (LMR) in patients undergoing hepatectomy and to compare it to established biomarkers including the neutrophil-to-lymphocyte ratio (NLR) and platelet-to-lymphocyte ratio (PLR). Medical records were retrospectively analyzed for 652 HCC patients undergoing hepatectomy at the Affiliated Tumor Hospital of Guangxi Medical University and the First People’s Hospital of Changde between April 2004 to April 2012. The correlation between the LMR and clinical variables were analyzed in Kaplan-Meier log-rank survival analysis and then multivariate Cox regression models trying to find relation with disease-free survival (DFS) and overall survival (OS). The area under the ROC curve (AUC) of the LMR(AUC:0.627) for predicting long-term survival was greater than that of the NLR(AUC:0.600) and the PLR(AUC:0.520).Multivariate analysis showed LMR to be an independent risk factor for OS (P = 0.002), and the NLR and PLR were not independently significant. Subgroup analysis also showed that LMR was significantly associated with poor DFS and OS in patients positive for HBsAg or with cirrhosis (both P < 0.001).Elevated preoperative LMR is an independently associated with poor OS and DFS in HCC patients following curative resection and appears to be superior to NLR and PLR.

## Introduction

Hepatocellular carcinoma (HCC) is one of the most common malignancies in the world and the third leading cause of tumor death^1–4^. Although hepatectomy has achieved significant improvements, the prognosis of patients with liver cancer is far from satisfactory, due to distant metastasis and tumor recurrence^5–8^. Recently, the use of inflammatory and molecular biomarkers to improve the prognosis of HCC has received increasing attention. However, there is still a lack of reliable, low-cost tumor markers that can be easily applied to clinic to predict prognosis.

There is more and more consensus that inflammation is associated with the development of malignant tumors and that persistent systemic inflammatory responses is involved with poor prognosis in many cancers^9–13^. One widely studied inflammatory markers originated from full blood count are the lymphocyte-to-monocyte ratio (LMR), the neutrophil-to-lymphocyte ratio (NLR) and the platelet- to lymphocyte ratio (PLR), which have been identified as prognostic biomarker in HCC^7,14–16^.

However, there are few studies to compare the ability of the LMR, NLR and PLR to predict long-term survival among patients with HCC undergoing liver resection with curative intent. Although Yang YT *et al*. have shown that lymphocyte to monocyte ratio and neutrophil to lymphocyte ratio are superior inflammation-based predictors of recurrence in patients with hepatocellular carcinoma after hepatic resection, they did not conduct survival analysis of over survival(OS)^16^.

In light of these, the present study aimed to compare the relative prognostic value of the preoperative LMR, NLR and PLR in disease-free survival(DFS) and OS in HCC patients with an attempt to clarify the optimal use of these markers.

## Materials and Methods

This research was approved by the Ethics Committee of the First People’s Hospital of Changde and the Tumor Hospital of Guangxi Medical University, and written informed consent was obtained from patients prior to surgery. All treatments were performed in accordance with relevant guidelines and regulations.

### Patients

All HCC patients underwent hepatectomy with curative intent at the First People’s Hospital of Changde City and the Affiliated Tumor Hospital of Guangxi Medical University between April 2004 and April 2012 were included in our study. The inclusion criteria were: no treatment for HCC before hepatectomy; initial hepatectomy with curative intent performed at the authors’ center; no other simultaneous malignancies; no coexistent hematologic disorders and no preoperative fever, so that the preoperative platelet count, neutrophil, lymphocyte and monocyte reflected normal baseline values; and no renal, cerebral, or cardiopulmonary dysfunction before hepatectomy.

### Diagnosis and definitions

Diagnose of HCC was based on the results of postoperative pathology. Hepatectomy with curative intent is defined as complete resection of all visible tumor and no residual tumor cells at surgical margins^17^. Preoperative NLR, LMR and PLR were determined within 7 d before surgery.

### Follow-up visits

All patients were followed up one month after liver resection and followed by a three-month interval in the first year, and then every 3–6 months in subsequent years, as appropriate. At each follow-up, serum AFP assay, ultrasound, serum biochemistry, chest X-ray and abdominal CT or MRI were performed.

### Statistical analysis

Statistical analysis was carried out with SPSS 19.0 (IBM, USA). Intergroup differences in continuous variables were compared by t-test or Mann–Whitney U test, as appropriate. While intergroup differences in categorical data were analyzed by two-sided χ^2^ test, Mann–Whitney U test or Fisher’s exact test, as appropriate. The area under the receiver operating characteristic (ROC) curve was calculated to measure the discriminatory power of the LMR, NLR and PLR as predictors of overall survival(OS). And an optimal cutoff value of 4.01, 2.78 and 99.5 corresponded to the maximum joint sensitivity and specificity on the ROC plot for LMR, NLR and PLR. Survival analysis was conducted to compare the over survival(OS) and disease free survival(DFS) rates using Kaplan-Meier survival curves with log-rank tests and Cox proportional hazard regression analyses, and p < 0.05 was considered statistically significant.

### Data sharing statement

Technical appendix, statistical code, and dataset are available from the corresponding author at cdsdyrmyy01@163.com. Participants gave informed consent for data sharing.

## Results

### Baseline characteristics of all patients

Of 854 consecutive patients who underwent hepatic resection with curative intent from April 2004 to April 2012 inclusive, 145 patients (16⋅9%) experienced other treatments before hepatectomy; 20 patients (2⋅3%) had other malignant tumors simultaneously; 25 patients (2⋅9%) had preoperative fever and coexistent hematologic disorders, and 12 patients (1⋅4%) had cardiopulmonary, cerebral or renal dysfunction before hepatectomy. After exclusion, 652 patients (76⋅3%) were enrolled in our study.

**Figure 1:**
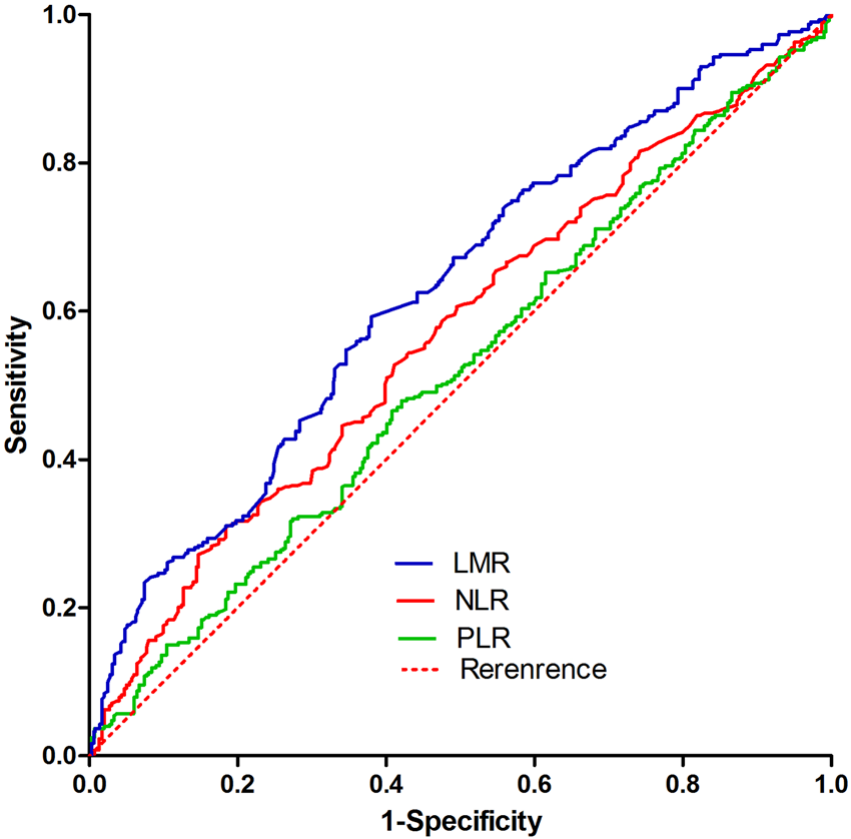
Receiver operating curve (ROC) for the preoperative LMR, NLR and PLR in predicting long-term survival with an optimal cutoff value of 4.01 (sensitivity:62.1 percent,specifiity:59.2 percent, AUC:0.627) for LMR, 2.78 (sensitivity:30.9 percent,specifiity:81.6 percent, AUC:0.600) for NLR, and 99.5 (sensitivity:47.9 percent, specifiity:57.9 percent, AUC:0.520) for PLR. AUC: (LMR, 0.656 vs. PLR, 0.600 P < 0.001).

Our study were consisted of 566 males and 86 females. Details of baseline characteristics of all HCC patients and separately in the low and high LMR groups can be seen in Table 1. ROC analysis found the optimal cutpoint for the LMR, NLR, and PLR were 4.01, 2.78 and 99.5, respectively (Fig. 1). The area under the ROC curve (AUC) of the LMR (AUC:0.627) for predicting long-term survival was greater than that of the NLR (AUC:0.600) and the PLR(AUC:0.520).

**Table 1:** Characteristics of HCC patients treated by resection.

Characteristic	LMR < 4.01 N = 341	LMR ≥ 4.01 N = 311	P
Gender, M/F	299/42	267/44	0.490
Age, yr	48.01 ± 11.5	46.9 ± 11.3	0.200
HBsAg			0.526
Negative	52	42	
Positive1	289	269	
Liver cirrhosis			0.529
No	60	49	
Yes	281	262	
AFP, ng/mL			<0.001
<400	134	209	
≥400	207	102	
BCLC stage			0.130
0 or A	175	178	
B or C	166	133	
Edmonson grade			0.411
I–II	116	268	
III–IV	73	195	
Child-Pugh class			0.533
A	322	297	
B	19	14	
Tumor number			0.010
Single	225	234	
Multiple	116	77	
Tumor size,cm	7.7 ± 3.7	6.1 ± 3.1	<0.001
Tumor capsule			0.172
Complete	129	134	
Incomplete	212	177	
Albumin, g/L	39.6 ± 4.2	40.5 ± 5.0	0.009
Platelet count, 10^9^/L	122.4 ± 65.4	89.0 ± 42.3	0.040
AST, U/L	49(34–72)	39(30–53)	<0.001
ALT, U/L	41(28.5–56.5)	39(28–56)	0.253
Total bilirubin, μmol/L	13.1(9.7–17.6)	13.4(9.2–17.4)	0.789
NLR			<0.001
<2.78	203	282	
≥2.78	138	29	
PLR			<0.001
<99.5	147	210	
≥99.5	194	101	

Data are mean ± standard deviation or median (25th–75th interquartile range) unless otherwise indicated.

Abbreviations: HBs Ag, hepatitis B surface antigen; AFP, alpha-fetoprotein; BCLC, Barcelona Clinic Liver Cancer; AST, aspartate aminotransferase; ALT, alanine aminotransferase; HCC, Hepatocellular Carcinoma.

A lower LMR was significantly associated with higher values of AFP, tumor number, tumor size, PLT and AST (all P < 0.05). On the other hand, A lower LMR was significantly associated with lower values of albumin (P < 0.05). The lower LMR was also strongly associated with both higher NLR (P < 0.001) and higher PLR (P < 0.001). Especially, high NLR was more likely in the low LMR group (40.5%) compared to both the overall cohort (25.6%) and those with high LMR (9.3%).

### Cox analyses of survival associated with LMR

Univariate analysis and cox proportional hazards regression were used to identify factors associated with OS and DFS and the detailed results were shown in Table 2.

**Table 2:** Cox proportional hazards regression to identify predictors of overall and disease free survival in HCC patients treated by resection.

	Overall Survival						Disease–free Survival					
	Univariable			Multivariable			Univariable			Multivariable		
	HR	95% CI	P	HR	95% CI	P	HR	95% CI	P	HR	95% CI	P
Male	0.919	0.685–1.234	0.576				1.027	0.756–1.396	0.863			
Age (≥60 years)	1.003	0.752–1.337	0.984				0.918	0.690–1.221	0.557			
LMR <4.01	1.763	1.421–2.187	<0.001	1.454	1.142–1.851	0.002	1.60	1.296–1.975	<0.001	1.266	1.007–1.593	0.044
Positive HBsAg	1.198	0.876–1.638	0.258				1.224	0.898–1.667	0.201			
Liver cirrhosi	1.192	0.895–1.586	0.229				0.983	0.751–1.286	0.899			
AFP ≥ 400 ng/mL	1.451	1.173–1.795	0.001	1.174	0.940–1.466	0.157	1.580	1.282–1.948	<0.001	1.389	1.121–1.722	0.003
BCLC stage B or C	1.852	1.590–2.158	<0.001	1.279	1.056–1.550	0.012	1.768	1.518–2.060	<0.001	0.926	0.681–1.259	0.624
Edmonson grade III–IV	1.324	0.789–2.089	0.203				1.245	0.823–1.765	0.324			
Child-Pugh class B	1.350	0.877–2.076	0.172				1.219	0.730–2.036	0.448			
Tumor number	2.075	1.673–2.574	<0.001	1.542	1.221–1.946	<0.001	2.109	1.700–2.617	<0.001	1.941	1.527–2.466	<0.001
Tumor size ≥ 5 cm	2.214	1.729–2.835	<0.001	1.544	1.160–2.056	0.003	1.850	1.465–2.335	<0.001	1.533	1.140–2.063	0.005
Tumor capsule	2.164	1.721–2.720	<0.001	1.812	1.421–2.310	<0.001	1.639	1.319–2.037	<0.001	1.371	1.095–1.716	0.003
Albumin ≥ 35 g/L	1.401	0.980–1.962	0.053				0.957	0.655–1.400	0.822			
Platelet count ≥ 100 × 10^9^/L	0.991	0.729–1.348	0.956				1.344	0.964–1.874	0.081			
AST ≥ 80U/L	1.445	1.096–1.905	0.009	1.156	0.868–1.539	0.321	1.278	0.965–1.693	0.088			
ALT ≥ 80U/L	0.861	0.610–1.216	0.395				0.942	0.678–1.309	0.721			
Total bilirubin ≥ 17.1 μmol/L	1.134	0.893–1.439	0.304				1.042	0.826–1.315	0.729			
NLR ≥ 2.78	1.762	1.405–2.210	<0.001	1.284	0.989–1.668	0.061	1.797	1.436–2.250	<0.001	1.506	1.165–1.947	0.002
PLR ≥ 99.5	1.299	1.054–1.601	0.014	0.877	0.692–1.112	0.279	1.338	1.089–1.645	0.006	0.990	0.788–1.243	0.930

Abbreviations: HBsAg, hepatitis B surface antigen; AFP, alpha-fetoprotein; BCLC, Barcelona Clinic Liver Cancer; AST, aspartate aminotransferase; ALT, alanine aminotransferase; HCC, Hepatocellular Carcinoma; HR = hazard ratio; CI = confience interval.

**Figure 2:**
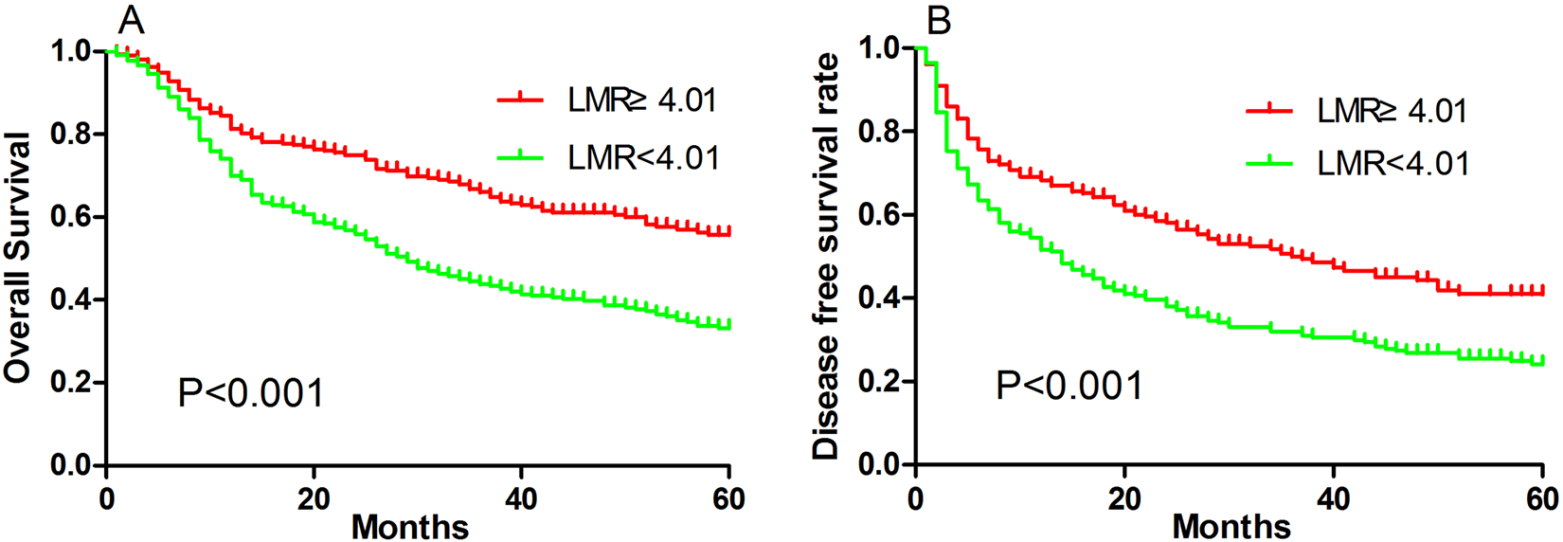
Kaplan-Meier survival curves showing overall survival (**A**) and disease-free survival (**B**) of hepatocellular carcinoma patients with high or low LMR.

Univariate analysis identified the following factors significantly associated with poor OS: LMR < 4.01, AFP ≥ 400 ng/mL, BCLC stage B or C, multiple tumors, tumor size ≥ 5 cm, incomplete tumor capsule, AST ≥ 80 U/L, NLR ≥ 2.78 and PLR ≥ 99.5. LMR < 4.01, AFP ≥ 400 ng/mL, BCLC stage B or C, multiple tumors, tumor size ≥ 5 cm, incomplete tumor capsule, NLR ≥ 2.78 and PLR ≥ 99.5 were also found to be significantly associated with poor DFS (Table 2, Fig. 2).

Multivariate analysis (Table 2) identified the following prognostic independent predictors of poor OS: LMR < 4.01, BCLC stage B or C, multiple tumors, tumor size ≥ 5 cm and incomplete tumor capsule. LMR < 4.01, AFP ≥ 400 ng/mL, B multiple tumors, tumor size ≥ 5 cm, incomplete tumor capsule and NLR ≥ 2.78 were also found to be significantly associated with poor DFS.

### Subgroup analyses associated with LMR

**Figure 3:**
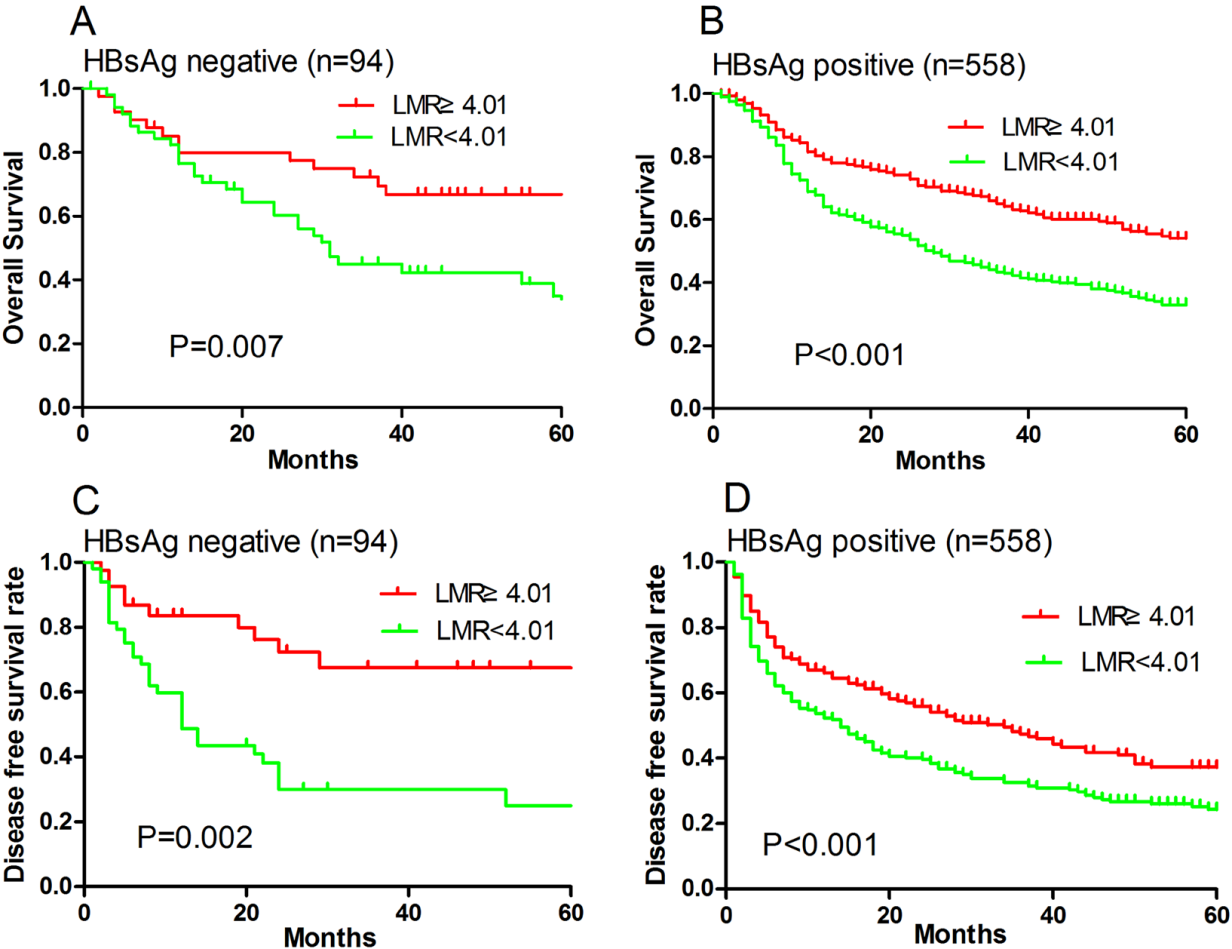
Overall survival (**A**,**B**) and disease-free survival (**C**,**D**) of HCC patients stratified by LMR according to (**A**,**C**) negative HBsAg and (**B**,**D**) positive HBsAg.

**Figure 4:**
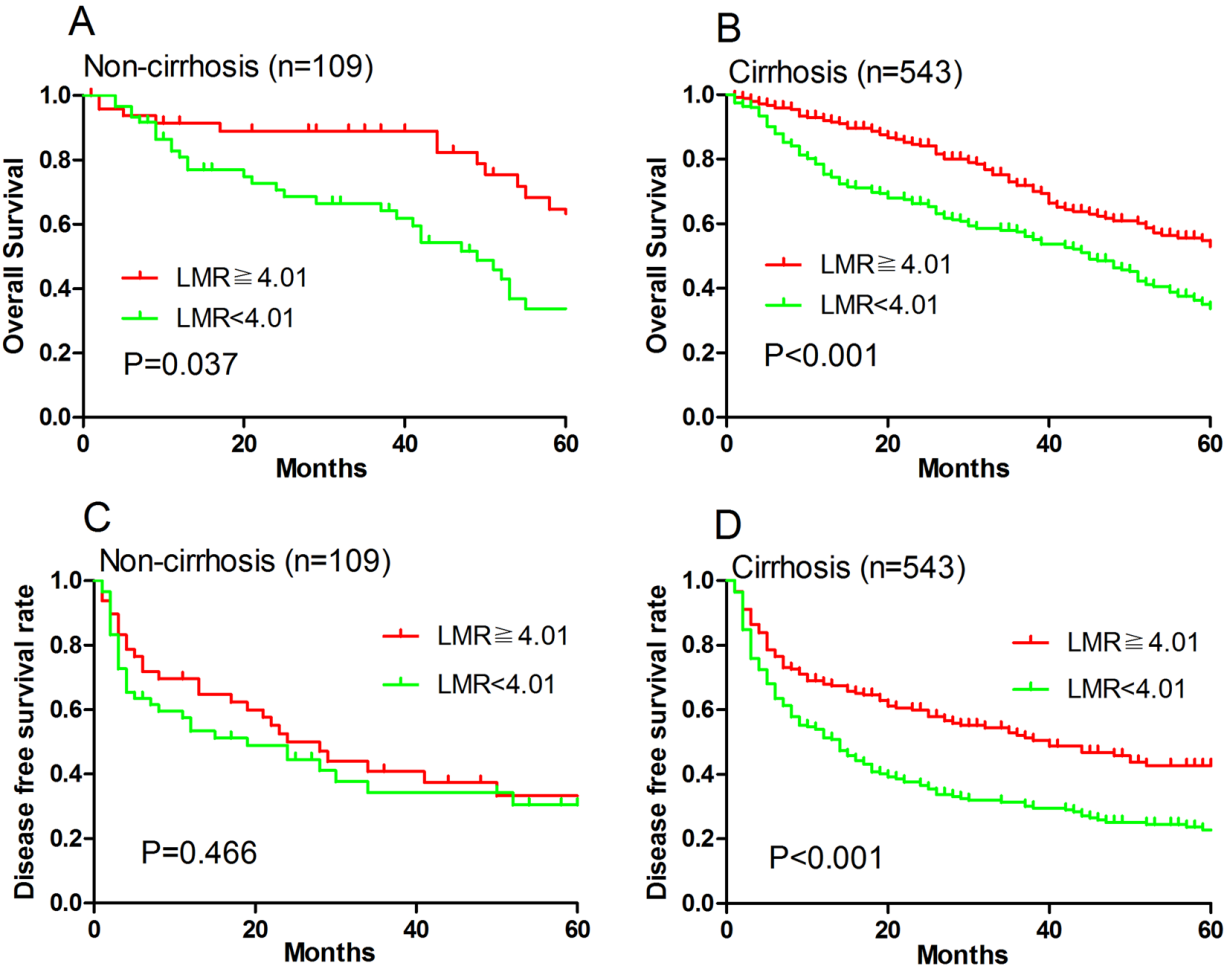
Overall survival (**A**,**B**) and disease-free survival (**C**,**D**) of HCC patients stratified by LMR according to (**A**,**C**) non-cirrhosis and (**B**,**D**) cirrhosis.

To clarify the subgroups of patients influenced by preoperative LMR, we grouped the patients according to HBV infection and liver cirrhosis, we discovered LMR was significantly associated with poor OS and DFS for patients with or without HBV infection (all P < 0.01; Fig. 3). LMR was also significantly associated with poor OS and DFS for patients with liver cirrhosis (both P < 0.001; Fig. 4B,D) but not for patients without cirrhosis (P = 0.466; Fig. 4C).

## Discussion

Our research implies that the preoperative LMR is an independent indicator of poor OS and DFS in HCC patients suffered curative resection. Furthermore, in the subgroup of patients positive for HBsAg or with cirrhosis, we found that the LMR was also significantly associated with OS and DFS.

We have also defined optimal cutpoints for LMR, NLR and PLR in the ROC analysis, with values of 4.01, 2.78 and 99.5, respectively. And we have found the area under the ROC curve (AUC) of the LMR (AUC:0.627) for predicting long-term survival was greater than that of the NLR (AUC:0.600) and the PLR(AUC:0.520). Furthermore, we have also found the LMR is a better indicator for long-term survival compared to NLR and PLR, both publicly known independent indicators of OS and DFS^7,14,18,19^. Before this research, studies had usually reported the NLR is superior to the PLR^16,20,21^. However, when studied with LMR, we found both NLR and PLR were not independent indicator for OS. What’s more, the cutpoints applied in my study for NLR and PLR of 2.78 and 99.5, respectively were consistent with previous researches^19,22^.

The mechanism of why decreased LMR should indicate poor survival remains unclear, but some researches have given some explanations. As far as we know, as early as the 1970s, these markers of lymphocyte components have been fully confirmed in the study in which lymphocytopenia were observed in patients with advanced tumor^23^. This has been one of the foundations of other established inflammatory markers, such as PLR and NLR. Although the correlation between monocytes and prognosis have been evaluated recently, only preliminary hypotheses have been made to explain why monocytes may indicate prognostic information. Here we put forward several possible mechanisms. First, previous study have found circulating monocytes may promote tumor growth and help tumor cells escape immune surveillance^24,25^. Second, derived from circulating monocytes, tumor-associated macrophages (TAMs) have been reported to be able to infiltrate into the HCC matrix, exerting activity including promotion of proliferation, metastasis, angiogenesis and immunosuppression^26–29^.

Although offering some new findings, this study has several limitations that need to be addressed. On one hand, since the value of LMR is dynamic during follow-up, we use only baseline values to predict future results, so we may miss a lot of information. On the other hand, due to the nature of the retrospective study, the potential choice bias exists.

In summary, our study suggests that the LMR is superior to the NLR and PLR, both established independent indicators of OS. However, a larger prospective study are needed to prove our researches.
